# Leaving no one behind: successful ageing at the intersection of ageism and ableism

**DOI:** 10.1186/s13010-023-00150-8

**Published:** 2023-11-24

**Authors:** Elisabeth Langmann, Merle Weßel

**Affiliations:** 1 Institute of Ethics and History of Medicine, University of Tübingen, Gartenstraße 47, Tübingen, 72074 Germany; 2 City of Hannover, Hannover, Germany

**Keywords:** Ageism, Ableism, Successful ageing, Intersectionality, Ethics, Mental health

## Abstract

**Background:**

The concept of ‘successful ageing’ has been a prominent focus within the field of gerontology for several decades. However, despite the widespread attention paid to this concept, its intersectional implications have not been fully explored yet. This paper aims to address this gap by analyzing the potential ageist and ableist biases in the discourse of successful ageing through an intersectional lens.

**Method:**

A critical feminist perspective is taken to examine the sensitivity of the discourse of successful ageing to diversity in societies. The paper analyzes how ageist and ableist biases can manifest in the ways we conceptualize ageing, drawing on examples in the context of mental health.

**Results:**

We argue that the conventional approach to successful ageing is limited in its ability to account for the experiences of people who have faced intersectional discrimination throughout their lives. Drawing on examples in the context of mental health, we explore among others the link between depression and disabilities. Furthermore, we shed light on the negative impact of ageist and ableist attitudes concerning the diagnosis and treatment of dementia.

**Discussion:**

We demonstrate how diversity is often overlooked in discussions of ageing well, and how ageist and ableist biases can manifest in the ways we conceptualize ageing. We argue that focusing solely on the health status as a means of achieving success fails to adequately counter ageism for all people. We further emphasize the role of structural factors, such as ageist attitudes, in shaping the experience of ageing and exacerbating health inequalities.

**Conclusion:**

Overall, our findings emphasize the need for a more nuanced and inclusive understanding of ageing and therefore an intersectional approach to conceptions of ageing well that recognizes and addresses the biases and limitations of current discourses. Thereby, this paper offers valuable insights into the complex intersections between age and disabilities from a bioethical perspective, highlighting the need for a more inclusive and intersectional approach to ageing.

## Introduction

Over the last decades, the growing emphasis on ageing well has led to the development and widespread use of concepts such as ‘successful ageing’, ‘productive ageing’, and ‘healthy ageing’ [1]. These concepts seek to define what constitutes desirable or ideal ageing and provide strategies for achieving it at both the individual and societal levels. In addition to promoting individual well-being, they also address societal challenges related to the demographic shift towards an ageing population. In this way, statements and assumptions are made about what kind of ageing is good and, at the same time, strategies are developed on how individuals and society can contribute to enabling, supporting, and implementing such ageing [2]. The focus is not only on individual well-being but also on societal challenges related to demographic change, such as the increase in the number of older people in society or in health care. However, such concepts do not only have a descriptive character, but also normative implications by illustrating possibilities for good ageing and considering what kind of ageing is most livable. Ehni et al. [3] identify the normative content under the following two aspects: First, they define criteria for measuring desirable outcomes. These can also be understood as evaluative reasonings for the kind of life older people *should* perceive as good. Second, they make recommendations about what individuals and society *should* do to achieve these outcomes. Consequently, gerontological conceptions of ageing well refer to an ageing process that can be optimized in certain desirable ways. In part because of these normative implications, such concepts are questioned. Especially the concept of ‘successful ageing’ by Rowe and Kahn [4] is widely used in scientific and political discourses, although it is also strongly criticized [5–8].

Building on this, in this paper, we adopt a critical feminist perspective towards the discourse of ‘successful ageing’ and aim to explore the potential inherent ageist and ableist biases revealed by an intersectional analysis of this concept. Intersectionality denotes a feminist theoretical framework that originated in the Black feminist movement in the United States in the 1970s and was academicized by Kimberlé Crenshaw in the late 1980s [9, 10]. It describes multi-categorical structural discrimination that is not visible in one-dimensional approaches to discrimination such as sexism or racism. Although rooted in the experiences of Black^1^ women and their experiences of sexism and racism, intersectionality has developed over the last three decades into a theory that allows for a critical engagement with different social categories and their intersections, such as gender, race, class, and age, and the power structures associated with them [15]. Intersectionality specifically addresses structural forms of discrimination while considering the impact of power relations on individuals. It has long been recognized that power relations in patriarchal or racist societies discriminate against individuals, such as women or Black people, and affect their well-being and social participation. However, the injustice of multi-dimensional discrimination, such as that experienced by Black women or older adults with disabilities, was basically invisible to both the affected individual and society until the term intersectionality was coined.

By analyzing the discourses of ageism, ableism, diversity, and intersectionality in the context of ‘successful ageing’, this paper examines carefully how this concept of ageing well is sensitive to the heterogeneity of older adults as a social group and how it can potentially contribute to ageist and ableist notions of ageing. Using examples from the context of mental health, we demonstrate how diversity is not adequately represented in discourses of ‘successful ageing’ and propose an intersectional perspective to address issues of ageism and ableism in discussions of 'good ageing’. Although the recent literature on medical ethics has begun to engage with intersectionality as a relevant approach to addressing multi-categorical inequalities and injustices in medicine and healthcare [16–19], there is still a significant research gap in the intersectional medical ethics approach to ageing and older age. However, particularly in the debates on successful ageing, the question arises of what successful ageing and a good life in old age mean for people who have experienced intersectional discrimination in relation to disabilities throughout their lives.

### Ageing within the context of successful ageing

Ageing and the changes associated with it are complex and multidimensional, and relate to various aspects of human existence, making it a highly interdisciplinary research topic. Ageing is a term for the continuing process of growing older, which is universal but without predictable outcomes. This process is strongly shaped by various factors, including the respective origin, gender as well as the relationships and environments experienced in the course of a lifetime [20]. In the context of health research, ageing, which shows a correlation between older age and diseases, can be described as a process that increases the likelihood of adverse changes in health. However, it needs to be noted that ageing is not a linear process, rather it is multidimensional and influenced by many factors, such as genetics or the social determinants of health, which can interact in a variety of highly complex ways [21]. This includes access to resources, social support networks, and opportunities for personal growth as significant determinants of health outcomes. It is therefore important to recognize that the ageing process is not predetermined but can be influenced by a combination of behavioral and structural measures. As some ageing individuals may experience a decline in mobility while maintaining cognitive abilities, others may experience the opposite. Moreover, different groups perceive older age in distinct ways, which further reflects the diversity of ageing. All this demonstrates that old age or being old, as well as the attributions associated with it, are also social constructs that appear differently depending on the perspective taken. While, for example, the socioeconomic status can strongly mold the individual experience of ageing [22] the process of growing older may also be perceived differently by gender or those who live with disabilities [23]. A reduction of age to a purely biological or chronological understanding must thereby be regarded as a truncated representation as it overlooks the distinct challenges and opportunities in the ageing process that vary among different members of social groups. Hence, generalizations concerning ageing, particularly across groups, are difficult and problematic. Despite recently increased efforts to highlight the different aspects of the ageing process and its positive implications, the prevalent deficit-oriented perspective towards older adults continues to persist, as was especially evident during the Covid-19 pandemic [24, 25]. From this standpoint, older adults are primarily seen as frail, dependent, and vulnerable, which is often based on stereotypes and prejudices that equate old age with illness [26, 27]. This view not only reveals a limited understanding of ageing, but also a narrow perception of the experience of illness and disabilities and reflects a form of ableism. Therefore, it is imperative to recognize the diverse experiences of ageing, to challenge ageist as well as ableist stereotypes, and to promote a more comprehensive understanding of ageing that acknowledges both its challenges and opportunities in regard to social position [26].

In contrast, successful ageing as a concept refers to an understanding of higher age that is marked by satisfaction, activity, independence, and overall refusal to accept traditional deficit-oriented narratives of older age. In the context of the growing number of older adults and the expected increase in political concerns about their economic impact, successful ageing influenced much of the research on ageing in the past. The breakthrough of this concept stems from an extensive interdisciplinary study led by John W. Rowe, which resulted in a large number of publications [5].

Overall, Rowe and Kahn argued that traditional research on ageing has emphasized average age-related losses and often neglected the heterogeneity among older people. Performing research on strategies for modifying the ageing process was aimed at supporting and facilitating the transition from usual ageing to successful ageing [5]. As a result of highlighting the heterogeneity of ageing and the positive aspects of higher age, successful ageing has been widely understood as a combination of empirical research and anti-ageist advocacy [28]. Later on, Rowe and Kahr introduced a more medical framework for the respective concept, that allowed differentiating between *usual* and *successful* ageing. According to them, successful ageing is based on the following three central components: (a) a low probability of illness, and thus disability; (b) high cognitive and physical performance; (c) active participation in life [29]. Thereby, it is emphasized that while the absence of illness is important for being successful in ageing, success can only be rounded off by active participation in life.

Consequently, the concept has often been criticized for its medicalization of older age and its ageist nature in particular for its narrow representation of health, functional capacity, and productive activity, as this terminology can support a negative perception of age(ing) – especially when (chronic) illness exist [28, 30–33]. In addition, its categorical construction of success, its implicit stigmatization of certain ageing processes and ways of being reflects a threat of ignorance. The central criticism in this context is directed towards the categorical objective of *good* ageing, which does not sufficiently take into account the many facets of ageing processes with its own priorities [28, 31], but implies normative expectations.

Furthermore, while successful ageing is primarily attributed to the respective individuals themselves, thus ignoring the diversity of health determinants, the idea of successful ageing also fails to consider the subjective perception of the ageing process of older people, as well as the social context in which they are situated [34]. Not only do the different realities of life go unremarked, but so do the health inequalities associated with them and their significant influence on meaningful opportunities. The limited perspective provoked by the dominant narrative of successful ageing fails to take into account the diverse experiences of ageing, as well as the particular challenges and barriers faced by people who are not only older but also members of racial, ethnic, gender or sexual minorities [35]. Meanwhile, due to the widespread awareness of successful ageing the perceived pressure to meet these criteria can create unrealistic expectations and feelings of inadequacy among older adults [36].

Calasanti [36] this highlights that a narrative of successful ageing coexists with the discourse of decline and generates pressure rather than displacing or reducing ageism, while neglecting to acknowledge older age as a stage with its priorities and foci [37]. In addition, different studies have shown that older people appreciate and imagine many different assets that perhaps are not being considered in the concept of successful ageing. These include self-acceptance, self-contentedness, social engagement and self-development in later life but also emotional and spiritual well-being, humor, and autonomy or pleasurable encounters with nature [3, 6, 38]. Thus, while successful ageing is portrayed as a marker for ageing well, the imagination of it can also be connected to heteronormative underpinnings [39]. However, the portrayal of successful ageing that takes place in the absence of illness is problematic for other reasons as well. Studies have shown that success in ageing is not necessarily dependent on objective health status, but rather that subjective perception and well-being play central roles [40]. In this regard, the literature often refers to the 'disability paradox', according to which people with severe and permanent limitations may assess their own quality of life as (very) good nevertheless, while this is perceived much more negatively by external observers [41]. Thus, success in ageing is not necessarily related to an objectively good and measurable state of health, but rather reflects a balance between psychological and physical well-being within a harmonious social environment [41].

If the heterogeneity of age(ing) is not sufficiently taken into account, this could lead to negative consequences for ‘unsuccessful’ older persons – e.g., in the form of less recognition, problematic political guidelines, or of health insurance contributions, such as higher deductibles [3]. In this way, this concept is, by definition, exclusionary, and its use and application pose risks of reinforcing ageism as well as ableism in its various forms. Consequently, the othering of usual or unsuccessful ageing encourages the disciplining of later life to the pursuit of able bodies and minds, while disregarding the experience of those dealing with illness and disabilities. Thereby this concept fails to confront the root causes of ageism, such as age relations and structural factors [33, 37]. Accordingly, ideas or perceptions of ageing well are problematic, as they explicitly or implicitly make normative claims, which can only partially correspond to the diverse experiences of ageing processes. If political measures align with such geriatric concepts, there is a risk that they will not only be ineffective but can also be harmful to individual well-being [3]. As a result, a narrow definition of health in older age can be interpreted as limiting and lacking in including the diverse experiences and realities of older individuals. But despite the evident limitations of this concept and criticism from various disciplines such as sociology or science and technology studies, the extent to which it is still used shows that many researchers, especially gerontologists, have become advocates that perpetuate this oversimplified idea of success in older age.

### Successful ageing at the intersection of ageism and ableism

#### Ageism

Ageism is defined as negative or positive stereotyping, prejudice, and/or discrimination against older adults based on chronological age or perceiving them as being ‘old’ or ‘older’ [42]. Thus, hostility towards older persons can have cognitive, affective, and behavioral components and can be implicit or explicit. Accordingly, ageism is strongly related to how we think, feel, and act towards older persons based on their chronological age or age classification [42]. As stated in the ‘Global Report on Ageism’ by the WHO, at least one in two people in the world experience ageism towards older adults, and one in three older people (in Europe) have been confronted with ageism, meaning this affects billions of people [20]. So individual, and societal perceptions of older age and being old play a key role in reinforcing stereotypes that can be both positive and negative [43]. As age is one of the first things we notice about persons, the ambiguity of using ‘old’ as a label is often overlooked in various deliberations. We tend to ascribe 'age-appropriate' characteristics and form opinions about ageing that are influenced by subjective and social attitudes, preferences, and so on. Such ideas, and thus the categorization of people in old age, are primarily based on prejudice, making them not only descriptive but also normative, and can lead to ageism [26].

In the context of health care, ageism may be associated significantly with poorer health and affect numerous health dimensions, such as physical and mental health as well as social well-being [44]. It includes the use of ageist language or even ‘elderspeak’ [20], as well as manifesting itself in a failure to take the health-related needs of adults in older age seriously [45] or even becoming visible in a refusal to perform certain medical procedures or tests on the basis of chronological age [46].

A common strategy for tackling ageism is to emphasize the heterogeneity of the health status of older age, which is also reflected in the concept of successful ageing, while overlooking older age as a position of disadvantage [33]. While highlighting this form of diversity can help to break down the homogeneous representation of older people, it also carries the risk of (even greater) differentiation between ageing in health, therefore successful, and ageing in illness, as either usual or even unsuccessful ageing. While this assertion of heterogeneity can break down prejudices and deficit-oriented views, it can also, as others have criticized, result in a dichotomy between successful ageing and unsuccessful ageing [28, 34]. This also carries the risk that those older adults to whom predominant ideas of ageing apply, e.g. because of their frailty or multimorbidity, confirm such prejudices. For example, older adults with chronic conditions or multimorbidity may be seen as confirming stereotypes about ageing rather than being recognized for their individual experiences and strengths. This means, that if arguments for the need to combat ageism are based solely on an understanding of diversity that primarily highlights the possibility of ageing in health, they will not be valid for *all* older adults. In particular, these arguments fail to include older adults living with illness and disabilities, leaving the inadequate treatment of older people with health limitations unchanged or even worse. Applying the diversity-of-ageing argument in light of health to the concept of successful ageing consequently implies that those who are considered successful in their ageing process should not be affected by ageism and serve as a normative standard against which other older adults are judged.

As a result, successful ageing does not only not replace the association of ageing and disabilities but urging an already disadvantaged group to exert more effort and improve self-care, the risk and impact of ageism remain the same or may even be exacerbated. Therefore, problematizing health-related limitations or disabilities of adults in older age reinforces the deficit-oriented perspective and the narrative of older adults as a burden on society. Focusing solely on individual responsibilities neglects the multidimensionality of health and the larger responsibility of society to ensure equal opportunities of participation for all and (unintentionally) justifies ageism towards those who are not successful. Consequently, using this argumentation to combat ageism is inadequate and carries the risk of ableism while the systematic disadvantage and exclusion of older adults is not considered [36].

#### Ableism

Similar to ageism, ableism refers to stereotypes, prejudices, and discrimination regarding people who live with disabilities or the perceived functional limitations of individuals [47]. This phenomenon reveals patterns of social oppression towards persons living with disabilities, and includes cognitive, affective, and behavioral aspects [48]. While ableism manifests itself in various forms such as inaccessible environments, barriers to participation in everyday life as well as unfavorable views or assumptions concerning disabilities, it treats disabilities primarily as a health problem rather than a social issue [49]. Based on this, the consequences are manifold, especially in the context of healthcare. Using data from the European Social Survey, a study by Branco et al. [50] highlights that people living with disabilities are one of the groups most affected by discrimination. Further, it is shown that facing ableism has a greater impact on health and well-being compared to discrimination based on memberships in other disadvantaged groups, such as those facing sexism or ageism. According to this, analogous to ageism, disparities exist in a number of areas such as screening and preventive services [51], diagnosis and treatment as well as interactions with medical professionals [52] and overall access to and experience in healthcare services [51, 53]. Thus, ableism and ageism intersect and share similar consequences.

Disability and old age are underrepresented categories in the context of intersectionality. Old age and age in general have been largely unrecognized in intersectional analysis as being a leading factor for structural discrimination though studies show that age in intersection with gender, race or socioeconomic status creates so far invisible structural disadvantages [16, 17]. The feminist movement has struggled to fully include women with disabilities in their politics, despite over one billion people living with disabilities globally, making them one of the largest minority groups in the world [1]. Despite attempts to include disability in the women’s movement, many women with disabilities have experienced ableism in the course of these efforts [45]. The specific discrimination that women with disabilities experience, such as being seen as inferior and having their bodies regulated by medical professionals, has not been addressed adequately and has been overlooked in feminist scholarship [54]. An intersectional approach to disabilities recognizes that it is constituted along the lines of gender, race, and class [54–56] and cannot be understood in isolation. Disability rights activists see ableism as a form of structural oppression, like sexism, racism, or classism [54]. Disabilities are not only a medical but also a social issue. The effects of ableism include the systematic removal of (people with) disabilities from all public spaces [54]. Despite this, there has been little systematic engagement by intersectional scholars with disabilities and ableism, which ends up excluding a wide range of structural discrimination based on norms of the able-bodied and therefore ableist norms.

#### The intersection of ageism and ableism in the context of mental health

The mental health of people in older age with disabilities is a prime example of the need for an intersectional approach. Although an increasing number of older individuals are projected to have mental health conditions, such as dementia and depression, it is yet to be taken seriously [57]. Despite advances in the fields of psychology and psychiatry, the Freudian approach, that older people lack mental flexibility, is still commonly adapted to address mental health disorders [58]. People at the intersection of old age, disability, and mental health issues face discrimination [59] and also belong to a group experiencing the highest suicide rates worldwide, older men being most affected [60, 61]. Thereby, in addition to the aggregation of diverse risk factors, such as loneliness, losses, or illness, the internalization of ageist narratives and prejudices about the value of life in older age also contribute significantly to this dynamic. Depression, for instance, is often viewed as a natural consequence of ageing while therapeutical concepts for older people are still scarce. However, depression can lead to disabilities in older age, or pre-existing disabilities can be worsened by depression [62]. As research suggests mental health issues in older age vary by gender, affecting women more often than men, while lacking data concerning nonbinary gender dynamics [63]. The strong stigma attached to mental illness in our society, along with ableist associations [64], leads to many older adults feeling ashamed when dealing with mental health issues, which can act as a significant barrier preventing them from seeking medical help [39]. However, many older adults with mental illness are members of multiple marginalized social groups, and focusing on stigma alone may not only overlook lived experiences but also limit the effectiveness of treatment options due to bias. For example, Black older people with depression face greater stigma and are less likely to seek treatment due to negative attitudes towards mental health treatment [65, 66]. Black women also have a higher prevalence of depression than white women [67]. However, their lifelong experiences of discrimination, racism, poverty, and violence also make them more likely to develop depression in old age [65]. Class and education are also closely linked to race. Poorer older poorer people with a non-white background are more likely to experience depression due to high-stress exposure over their lifetime as well as a lack of psychosocial resources [68]. Race and relatedly class and education are leading intersecting categories that make it more likely for older people to become depressed in old age but also lower their chances for adequate help due to the intersectional discrimination they experience throughout their lifetime which accumulates in old age.

The categories of age, disability, and mental health are strongly intertwined, and the respective discrimination is based on ageist and ableist understandings of mental health that impact the treatment and quality of life of older people with disabilities who are experiencing depression. The interplay of depression in older age with disabilities [69, 70] is further exacerbated by mental health inequities driven by multifactorial discrimination, wherein individuals experience discrimination based on multiple social identities [71]. When people in older age live with depression, the risk of developing co-morbidities rises, for example, for major cognitive impairment or severe chronic illnesses, with the consequence of a decline in the overall health status [62]. Thereby discrimination due to gender, race, socioeconomic status, sexual orientation, or other identity markers such as disabilities is a significant predictor for high depression scores [71], with far-reaching consequences. Studies have shown, for instance, that there is a link between depression in earlier life and the development of dementia in later life [45, 46]. Additionally, depression in later life can be a risk factor for developing dementia in the near future [72]. Older people with depression and cognitive impairment often face multiple health challenges and complex medical needs, yet diagnosis and treatment are still scarce [73].

Furthermore, as dementia is understood as a disruption of a potentially successful ageing future and is also positioned as a threatening existence by society [37, 74], the relationship between ageism and dementia is strongly intertwined with ableism. Although dementia is not an inevitable part of ageing, the prevalence of the disease strongly increases with age [75] and affects a diverse group of older adults differently. For instance, while three in five people with Down syndrome in the group of older adults develop dementia, only about three percent of the general population receives a diagnosis of dementia [76]. Furthermore, the diagnosis is closely intersected with race and education [77]. It is well known for a while that a higher level of education can be a factor in the development of dementia. However, due to social injustice, non-white people often have a lower education than white people. Furthermore, non-white people from various racialized groups are more likely to develop dementia in old age than white people [78]. Additionally, especially Black and Hispanic people in particular face delays in diagnosis, and their dementia is often more advanced, by the time they receive a diagnosis, which affects treatment options [79]. Studies have further indicated that the diagnosis of dementia is based on racist prejudices and discrimination, for example that limited language skills and a lower levels of education can lead to false positive test results [17]. Race, education, class, and age are again closely intertwined and strongly intersecting factors in the development of dementia in old age and in this way adding disability as a further intersecting category to the portfolio of marginalized people.

As there is currently no cure for dementia, the fear of developing this condition is often accompanied by negative emotions, including humiliation, shame, and disgust, and is therefore strongly stigmatized [75]. It is argued that the rate of early diagnoses is quite low because of the negative perception of dementia, but also the willingness to seek diagnosis is negatively influenced [75]. Moreover, ageism, characterized by negative stereotypes about the cognitive abilities of older adults, can further impede a timely diagnosis of dementia. These deficit-oriented stereotypes can act as a barrier to receiving proper care and treatment. As outlined by Evans [75], there is a significant lack of support for people with dementia after diagnosis, including access to information and education, as well as a failure to provide beneficial treatments due to misconceptions and misjudgment by some health professionals. This often results in a higher prevalence of undiagnosed and preventable co-morbid conditions, which leads to disparities in health outcomes, including faster deterioration in daily functioning, reduced quality of life, and earlier death [75]. These discriminatory occurrences in healthcare settings may also be influenced by widespread ageist perceptions and the previously described negative effects on the healthcare and well-being of adults in older age [44]. While persons with dementia often experience intersectional discrimination that arises from both ageism, dementia-related ableism and in relation to previous categories such as gender and race, there is one crucial difference between those two forms: While ageism is often associated with negative views and narratives of ageing, it can also be accompanied by positive stereotypes such as becoming wiser with age [80]. In contrast, it is difficult to find positive attributes commonly associated with dementia. This lack of positive stereotypes may contribute to the social exclusion and stigma experienced by people living with dementia [75]. This is especially prevalent in racialized groups due to culturally different understandings of ageing but also of gender, for example masculinity [17]. This highlights the need for a critical and comprehensive approach to dementia that recognizes and addresses the unique challenges faced by older adults with cognitive impairment.

While well-intentioned, the concept of successful ageing also results in picturing an unsuccessful ageing process that can negatively impact the self-esteem of older adults dealing with mental health issues and contribute to ageist and ableist attitudes towards mental health. In addition, it does not take into account the diversity of the experiences of older adults, e.g., including adults who age with learning disabilities, whose ageing process has thus been largely invisible [81]. However, a study has shown that people at the intersection of old age, intellectual disability, and cognitive impairment face significant challenges in receiving adequate care [82]. As this group of persons is often considered less healthy and productive throughout their lives, they are frequently excluded from discourses about successful ageing, contributing to their ageing process remaining largely invisible [58, 60]. From a younger age, people with learning disabilities struggle to be taken seriously and have their needs adequately met. Often they experience infantilization. Women with learning disabilities in particular experience surveillance and restrictions by institutions and family [83]. Additionally, people with learning difficulties face different and earlier disabilities which are associated with old age [84]. For example, they may be less mobile due to their disability and need walking aids. As a result. they may become frail earlier than the average ageing person. However, especially geriatric medicine is not prepared for this diversity in ageing and often sees disability as a consequence of frailty in old age rather than frailty as a consequence of disabilities in younger age, leading inadequate care for older people with learning disabilities [84]. There is very little research on older racialized people with learning disabilities and their experiences of ageing. Yet, some studies indicate that, for example younger Black people with learning disabilities face severe discrimination in their daily lives, which is likely to accumulate over a lifetime as a result of their race and disabilities, combined with the poverty they often experience [85].

## Discussion

As concepts of ageing well have evolved over the years, designations such as ‘successful ageing’ have shaped the scientific and societal discourse on what constitutes *good* ageing. In the context of successful ageing, being successful implies achieving a low probability of disease, maintaining high levels of cognitive and physical performance, and actively participating in various aspects of life [29]. However, the nature of ageing is complex and multidimensional, with various factors, such as genetics, social determinants of health, as well as personal behaviors, influencing the outcome [2, 24]. By establishing a desirable way of ageing and thereby defining criteria for measurable results, recommendations are primarily made on how individuals can achieve them. In consequence, normative implications on how to grow older are defined implicitly or at least can be derived directly.

Especially the importance of the manifold impact originating in social and structural determinants of health is often disregarded or overlooked. Despite recent studies demonstrating the significant impact of social determinants on well-being [86, 87], this is not reflected in the current state of healthcare, as evidenced by the lack of standardization and screening tools to track these variables, and inadequate healthcare-based solutions for core problems [88]. Furthermore, research has shown that structural determinants, such as governance, social and public policies, and social and cultural values, play an important role in shaping an individual’s socioeconomic position, which in turn affects intermediate determinants of health, such as material circumstances and psychosocial and behavioral factors [89]. It is important to highlight that such determinants are often beyond the control of individuals [90]. A comprehensive understanding of the complex interactions between these factors is therefore crucial to improving health outcomes for both individuals and populations. In light of the absence of considering these influences in the concept of successful ageing, there has been criticism in recent years regarding its narrow focus on health and productive activity. Additionally, it has been criticized for its failure to account for subjective perceptions of health and social contexts [34]. It can be deduced that this well-known and widely used concept does not adequately consider the heterogeneity and various realities of older adults as a social group.

Building on this criticism, our approach of addressing the concept of successful ageing from a critical feminist perspective reveals (potential) inherent ageist and ableist biases. By analyzing the concept from an intersectional perspective, the perception of ageing successfully is questioned through the lenses of ageism and ableism as two widely spread phenomena of prejudice, stereotypes, and discrimination in older age. It is noteworthy that both ageism and ableism can have negative impacts on health and well-being [44, 50]. In this regard, we aim to emphasize the risk of perpetuating existing prejudices against certain groups of older adults when focusing on their ability to define the ageing process through individual lifestyle choices. In turn, this unintentionally draws an oversimplistic picture of ageing, disregarding the impact of structural inequalities and the accumulation of social factors over a lifetime, one example presents itself in the significant negative effect of lower socioeconomic status on health and consequently on ageing and disabilities [91].

Since the concept of successful ageing uses the heterogeneity of older age as an argument for challenging deficit-oriented views of older age, it uses the (common) strategy of emphasizing the heterogeneity of health in older age to combat ageism. This can potentially unravel the narrative that equates older age with illness, while still (further) promoting the dichotomy between successful and unsuccessful ageing. Considering this, despite the aim of highlighting the wide range of health statuses of adults in older age, the concept of successful ageing also unintentionally problematizes ageing with health limitations by excluding such ways of growing older from being deemed successful. This is further explored with the examples of the intersectionality of ageism and ableism in the context of health care, highlighting the stigmatization and discrimination faced by older adults with disabilities and its impact on their medical treatment and overall quality of life. Thereby, this paper addresses the link between depression and disabilities, while also shedding light on how ageist and ableist attitudes can negatively affect the diagnosis and treatment of dementia. We demonstrated that stigmatization and discrimination often accumulate over the course of a lifetime, and that people who have experienced discrimination on the basis of gender, class or race during their lives face even greater disadvantages in older age, which are not adequately recognized in successful ageing discourses. In this regard especially, we criticize the concept of successful ageing for its limited understanding of the diverse experiences of older adults with disabilities.

Although Rowe and Kahn developed the concept to counter ageism [28, 29, 92], the intersectional perspective taken here uncovers that such argumentation is suitable for combating ageism only for those who age according to the propagated understanding of health. Also, this inherent problematisation of ageing, within the concept even of those who are defined as ageing 'usually', reinforces two predominant narratives – the vulnerability narrative and the burden narrative – through the concept applying at least to all those who are not interpreted as successful in their ageing processes. In addition, such an understanding contributes to the view that disabilities are understood as being unhealthy, while being successful in choosing a healthy life(style) is interpreted as a marker for ageing well. All this reflects and propagates not only a narrow view of health but also of ageing. Based on this, we argue that applying the diversity-of-ageing argument solely in relation to health status as a means of achieving successful ageing is inadequate to combat ageism for *all* people. Rather, while overlooking the intersectionality of health, this approach carries the risk of ableism – as we outline in the described examples. This means that the conventional narrative of successful ageing perpetuates ageist and ableist assumptions, whereby persons, who age with disabilities, or those, who acquire disabilities in later life, are seen as incapable of successful ageing. In addition to this construction of success, the stigmatization of certain ageing processes and ways of being is a form of ageism and ableism, as it reflects a lack of understanding and appreciation for the diversity of human experience. In this respect, the concept of successful ageing needs to be revised in a critical manner. This ought to be directed towards the misleading notion that the ageing process is largely controlled and influenced by individual choices, which, as described above, ignores the impact of structural factors, also including different forms of marginalization, that can affect the experience of ageing and exacerbate health inequalities which can be highlighted through an intersectional lens.

In contrast to that, an intersectional approach to ageing means taking into account the multidimensionality of health and the diversity of adults in later life as well as the impact of power structures on good ageing. This includes recognizing there is no universal definition of ageing well; rather, it is of central importance to consider the diverse experiences and backgrounds of adults in older age. This approach recognizes that factors such as race, gender, socio-economic status, and disabilities can all influence individual experiences of ageing as well as access to resources that are essential to health and well-being. One model for such a perspective is the work of Meika Loe on comfortable ageing, which offers an alternative that focuses primarily on the subjective definition and accessibility of ageing well [93]. By considering the intersecting identities and experiences of *all* older adults, we can develop a more nuanced and inclusive understanding of ageing well, that recognizes and values the entire diversity of older populations – apart from health status – leaving no one behind.

## Conclusion

In this article, we examine the widely used concept of successful ageing developed by Rowe and Kahn and its implications for societal views of ageing and the ageing process. We build on the critique that successful ageing, which emphasizes individual agency and control, fails to consider structural factors that strongly influence growing older. We argue that this is particularly problematic because it implies that ageing is predominantly within the control and responsibility of the individual, disregarding the multidimensionality of health and its various social determinants. The intersectional perspective taken in this article has revealed the potential inherent ageist and ableist biases in the concept of successful ageing. By analyzing the concept from this perspective, it has been shown that it may perpetuate existing prejudices against certain groups of older adults and may exclude ways of growing older from being considered successful. This means the concept wrongfully excludes particular individuals and can perpetuate ageism and ableism, leading to health inequalities and social exclusion. However, given the extent to which this concept is still used, researchers become advocates who uphold this oversimplified idea of success in later life. Therefore, a critical revision of the concept of successful ageing that takes the impact of these factors into account is necessary. By adopting an intersectional approach to ageing, we can acknowledge and address the diversity of experiences of ageing and combat ageism as well as its intersections with ableism. This is essential to ensure that everyone, regardless of their age or ability, can be supported in ageing well – leaving no one behind. We, moreover, draw attention to the need for recognizing that structural factors such as ageism and ableism can have a significant impact on the experience of ageing and can exacerbate health inequalities. Drawing on the described examples we discuss the intersectionality of ageism and ableism in healthcare, highlighting the negative impact of discrimination and stigmatization of older adults with disabilities. As a result, we investigate the relationship between depression and disabilities and shed light on the negative impact of ageist and ableist attitudes on the diagnosis and treatment of dementia. This paper, therefore, provides valuable insights into the complex intersections between ageing and disabilities from a bioethical perspective. Continuing the discussion around concepts of ageing well requires, besides further research, a multi-faceted approach that includes engaging with marginalized groups and developing inclusive policies. By taking an intersectional approach, we can collaborate to promote a more inclusive and equitable ageing experience for every individual.

## Data Availability

Not applicable.

## Footnotes

1 We use the term Black to refer to people who experience racism on the basis of their skin colour or African descent. The term has its origins in anti-racism discourses in the United States, but is increasingly being used in European contexts to highlight racism based on skin colour or descent experienced by Black people in Europe. Its use draws critical attention to longstanding silence about racism in European cultures, as well as to current decolonial and critical whiteness discourses. For more information see  [11–14].
